# A machine learning model integrating radiomics and clinical factors to predict recurrence risk after rehabilitation in lumbar disc herniation

**DOI:** 10.1097/MD.0000000000050454

**Published:** 2026-09-11

**Authors:** Yanhong Qi, Yifei Feng, Xiaoyue Xing, Zhe Wang, Yuwei Yang, Lin Zhang, Weiguo Yu

**Affiliations:** a Medical Department, Beidaihe Rest and Recuperation Center of the Joint Logistics Support Force, PLA, Beidaihe City, Hebei Province, China; b Department of Medical Imaging, Beidaihe Rest and Recuperation Center of the Joint Logistics Support Force, PLA, Beidaihe City, Hebei Province, China; c Special Care Department, Beidaihe Rehabilitation and Recuperation Center, Joint Logistics Support Force, PLA, Beidaihe City, Hebei Province, China.

**Keywords:** lumbar disc herniation, machine learning, predictive model, radiomics, recurrence, rehabilitation

## Abstract

This study aimed to construct and internally validate a machine learning model integrating radiomics features and clinical factors to predict post-rehabilitation recurrence risk in patients with lumbar disc herniation (LDH). In this single-center retrospective cohort study, 260 adults aged 18 to 70 years with LDH who completed standardized rehabilitation were analyzed (78 with recurrence and 182 without recurrence). Radiomics features were extracted separately from sagittal T1-weighted, sagittal T2-weighted, and axial T2-weighted magnetic resonance imaging, standardized, concatenated across sequences, and reduced using least absolute shrinkage and selection operator regression. Three logistic regression models were developed: radiomics-only, clinical plus rehabilitation, and combined models. A stratified 70/30 split provided internal validation; receiver operating characteristic curves, calibration analysis, decision curve analysis, and risk stratification were used to evaluate performance. A separate multivariable logistic regression analysis was used to identify clinically interpretable predictors. Age, body mass index, disc height index, improvement in the Oswestry Disability Index, diabetes, Modic changes, and poor rehabilitation adherence differed significantly between the recurrence and non-recurrence groups (all *P* < .05). The combined model demonstrated the best predictive performance, with an area under the curve (AUC) of 0.85 (95% confidence interval: 0.78–0.91), outperforming the radiomics model (AUC = 0.78) and the clinical model (AUC = 0.76). The calibration curve showed strong agreement between predicted and observed recurrence, and decision curve analysis indicated superior net clinical benefit across a wide range of thresholds. Based on predicted probabilities, patients were stratified into low- (<20%), intermediate- (20%–40%), and high-risk (>40%) groups, with recurrence rates of 15.0%, 30.0%, and 66.0%, respectively (*P* < .001). Multivariable logistic regression identified disc height index (odds ratio [OR] = 1.80), Oswestry Disability Index improvement (OR = 0.65), poor rehabilitation adherence (OR = 2.50), diabetes (OR = 2.10), and Modic changes (OR = 1.90) as independent predictors of recurrence. Integrating multisequence weighted magnetic resonance imaging radiomics with clinical and rehabilitation variables improved internal prediction of post-rehabilitation recurrence in LDH. The combined model may support risk-adapted rehabilitation intensity and follow-up, but it requires prospective, multicenter external validation before routine clinical use.

## 1. Introduction

Lumbar disc herniation (LDH) is one of the most common degenerative spinal disorders leading to low back pain, radicular symptoms, and lower-limb dysfunction. Its global prevalence ranges from 1% to 3% and has shown a progressive upward trend in recent years.^[1,2]^ Following decompression surgery or structured conservative treatment, patients with LDH typically undergo standardized rehabilitation programs aimed at restoring neurological function, improving trunk stability, and enhancing overall quality of life.^[3]^ However, despite continuous refinement of rehabilitation strategies, approximately 20% to 40% of patients experience symptom recurrence, including worsening pain, functional decline, or recurrent herniation on imaging.^[4,5]^ Recurrence not only increases healthcare utilization but also adversely affects long-term functional outcomes, making early identification of high-risk individuals a critical component of LDH management.

Multiple studies have explored recurrence-related risk factors from clinical, biomechanical, and imaging perspectives. Existing evidence suggests that advanced age, elevated body mass index (BMI), intervertebral disc degeneration, decreased disc height, Modic changes, diabetes, and poor rehabilitation adherence may contribute to posttreatment recurrence.^[6–9]^ Nevertheless, many conventional clinical indicators rely on subjective assessment, and the complex interactions among variables limit the development of accurate and generalizable risk-prediction models. Although magnetic resonance imaging (MRI) serves as the primary imaging modality for LDH by clearly visualizing disc morphology, endplate alterations, and neural structures, traditional MRI interpretation predominantly depends on qualitative assessment. Such approaches may overlook subtle structural variations and therefore restrict the predictive utility of MRI in recurrence assessment.^[10]^

The rapid advancement of radiomics has opened new avenues for disease prediction. Radiomics extracts high-dimensional quantitative features from standard MRI sequences, capturing texture, shape, and intensity patterns that may be imperceptible to visual assessment. This technique has shown promise in spinal degeneration and outcome prediction.^[11–13]^ A recent MRI-radiomics study predicted recurrent L4 to L5 disc herniation, but radiomics evidence specifically addressing recurrence after a completed rehabilitation program remains scarce. Existing LDH recurrence models have generally emphasized conventional clinical or visually assessed imaging variables, whereas rehabilitation adherence and multisequence quantitative imaging have rarely been integrated within a single clinically evaluated framework.^[13–15]^ This gap limits individualized post-rehabilitation surveillance and rehabilitation planning.

Accordingly, this study aimed to develop and internally validate a machine learning model that integrates multisequence MRI radiomics, clinical factors, and rehabilitation adherence to predict recurrence after rehabilitation in patients with LDH. Its original contribution is the joint modeling of tissue-level quantitative imaging and patient-level recovery behavior, followed by comparison with single-domain models and evaluation of calibration, decision-analytic utility, and clinically usable risk strata.

## 2. Methods

### 2.1. Study design and reporting

This single-center retrospective cohort study was conducted at the Beidaihe Rest and Recuperation Center of the Joint Logistics Support Force, China (2025-JLSF-KS047). Medical records and pre-rehabilitation MRI examinations acquired between January 2020 and December 2023 were reviewed. The study was approved by the institutional Ethics Committee of the Joint Logistics Support Force; the committee waived written informed consent because only retrospective data were analyzed. All procedures conformed to the Declaration of Helsinki. In brief, eligible adults with MRI-confirmed LDH who completed a standardized rehabilitation program were followed for at least 12 months, and clinical, rehabilitation, and multisequence MRI variables were used to develop and internally validate recurrence-prediction models.

### 2.2. Study population and eligibility criteria

Patients were included if they met all of the following criteria: age 18 to 70 years; MRI-confirmed single- or 2-level LDH; completion of at least 12 weeks of standardized rehabilitation, including core-muscle training, neural mobilization, manual therapy, strengthening exercise, and postural correction; at least 12 months of follow-up; and complete pre-rehabilitation MRI and key clinical data. Exclusion criteria were the following: previous lumbar surgery; MRI quality inadequate for radiomics or metal artifacts; spinal tumor, infection, severe stenosis, deformity, or another neurological or orthopedic disorder that could independently explain the symptoms or materially interfere with rehabilitation; severe uncontrolled medical or psychiatric illness that prevented adherence or reliable follow-up; and missing key clinical, imaging, or outcome data. The final analytic cohort comprised 260 eligible patients: 78 with recurrence and 182 without recurrence. Because complete imaging and 12-month outcome data were eligibility requirements, no participant was excluded or lost after entry into the analytic cohort. The archived dataset did not retain a count-by-reason screening log for patients assessed before eligibility; this reporting limitation is acknowledged below.

Sample-size adequacy was evaluated for the prespecified 5-parameter clinical logistic regression component using an events-per-parameter sensitivity analysis for a binary outcome. With a recurrence proportion of 30%, a minimum of 10 events per parameter required 5 × 10/0.30 = 166.7, rounded up to 167 participants; a more conservative target of 15 events per parameter required 5 × 15/0.30 = 250 participants. The available cohort of 260 participants included 78 recurrence events, corresponding to 15.6 events per parameter, and therefore exceeded the conservative 250-participant target. In the 70% development subset, approximately 55 recurrence events were available, corresponding to approximately 11 events per parameter. This calculation supports the 5-parameter clinical logistic regression component only; it does not by itself establish sample-size adequacy for high-dimensional radiomics, for which least absolute shrinkage and selection operator (LASSO) shrinkage, internal validation, and subsequent external validation remain necessary. Because this was a retrospective cohort including all eligible patients during the study period, the calculation is reported as a sample-adequacy assessment rather than a prospective null-hypothesis power calculation.

### 2.3. Variable definitions and clinical data collection

Demographic and clinical data were obtained from electronic records and follow-up notes. Variables included age, sex, BMI, smoking history, diabetes, herniation level, disc height index, Modic changes, baseline Oswestry Disability Index (ODI), post-rehabilitation ODI, and rehabilitation adherence.

ODI was scored across 10 domains from 0 to 5 and converted to a 0 to 100 percentage, with higher values indicating greater disability.^[16]^ ODI improvement was calculated as (baseline ODI − post-rehabilitation ODI)/baseline ODI × 100%. Rehabilitation adherence was the percentage of prescribed sessions completed, verified from attendance and rehabilitation records. Poor adherence was operationally defined a priori as <70%, representing non-completion of at least 30% of prescribed sessions; because no universally accepted LDH-specific cutoff exists, this threshold should be interpreted as a pragmatic study definition rather than a validated biological boundary.

Disc height index was obtained from mid-sagittal MRI by normalizing intervertebral disc height to adjacent vertebral-body dimensions to reduce the influence of patient size, following the principle of standardized disc-height measurement.^[17]^ Modic changes were classified on T1-weighted imaging (T1WI) and T2-weighted imaging (T2WI) as type I (T1 hypointense/T2 hyperintense), type II (T1 hyperintense and T2 iso- or hyperintense), or type III (hypointense on both sequences); any type was coded as present for the primary analysis.^[18]^ Recurrence was defined as renewed low back and/or radicular pain that affected daily activities after initial improvement and was accompanied by MRI-confirmed recurrent herniation at the same or an adjacent level during 12-month follow-up.

### 2.4. MRI acquisition and radiomics feature extraction

All MRI examinations were performed using the same 3.0-T scanner (Magnetom Skyra, Siemens Healthineers, Germany). Patients were positioned supine using a standard lumbar phased-array coil. The imaging protocol included sagittal T1WI, sagittal T2WI, and axial T2WI, which were used for radiomics analysis.

Imaging parameters were as follows: sagittal T1WI: repetition time (TR) 500 to 650 ms, echo time (TE) 8 to 12 ms, slice thickness 3.0 mm, interslice gap 0.3 mm, field of view (FOV) 280 × 280 mm, matrix 320 × 256, echo-train length 9; sagittal T2WI: TR 2800 to 3500 ms, TE 100 to 120 ms, slice thickness 3.0 mm, gap 0.3 mm, FOV 280 × 280 mm, matrix 384 × 320, echo-train length 15; and axial T2WI: TR 3000 to 4000 ms, TE 90 to 110 ms, slice thickness 4.0 mm, gap 0.4 mm, FOV 180 × 180 mm, matrix 320 × 256.

To ensure radiomics reproducibility, all images underwent standardized preprocessing: gray-level normalization to μ ± 3σ; resampling to isotropic 1.0 × 1.0 × 1.0 mm^3^ voxels; voxel reconstruction using a B-spline algorithm; and N4ITK bias-field correction to reduce intensity nonuniformity.

All images were exported in the Digital Imaging and Communications in Medicine format and converted to Nearly Raw Raster Data format for region-of-interest (ROI) segmentation and feature extraction.

Radiomics features were initially extracted separately from sagittal T1WI, sagittal T2WI, and axial T2WI. After feature standardization and LASSO-based selection, only the most informative features with nonzero coefficients were retained for final model construction. Therefore, although all 3 sequences were analyzed at the extraction stage, the final radiomics signature consisted only of the selected features that contributed most strongly to model performance.

### 2.5. MRI preprocessing, and ROI assessment

Radiomics features were first screened using LASSO regression with 10-fold cross-validation to determine the optimal penalty parameter λ. Features with nonzero coefficients were retained for model development.

Three predictive models were constructed: a radiomics model; a clinical + rehabilitation model; and a combined model integrating radiomics features, clinical variables, and rehabilitation adherence.

All models were based on logistic regression. The dataset was randomly divided into a training set (70%) for model construction and a validation set (30%) for performance evaluation. Hyperparameters were tuned using grid search.

Before feature extraction, images from each sequence underwent the same preprocessing pipeline: gray-level normalization to μ ± 3σ, resampling to 1.0 × 1.0 × 1.0 mm^3^ isotropic voxels using B-spline interpolation, and N4ITK bias-field correction. These operations reduced intensity and voxel-size heterogeneity while preserving the 3 sequences as separate feature sources. Digital Imaging and Communications in Medicine images were converted to Nearly Raw Raster Data format. ROIs covering the target herniated disc were manually delineated, and a second observer verified the contours; disagreements were resolved by consensus. Manual segmentation was used consistently across sagittal T1WI, sagittal T2WI, and axial T2WI, but its observer dependence remains a limitation.

### 2.6. Sequence-specific radiomics extraction and feature fusion

The radiomics workflow comprised 5 explicit steps: features were extracted separately from sagittal T1WI, sagittal T2WI, and axial T2WI; features were labeled by sequence to preserve their source; each feature was standardized using parameters derived from the training set, and the same transformation was applied to the validation set; the 3 standardized sequence-specific feature matrices were concatenated at the patient level; and LASSO logistic regression with 10-fold cross-validation selected features with nonzero coefficients from the fused matrix. Thus, no images were averaged across sequences, and feature fusion occurred before model fitting but after sequence-specific extraction and standardization.

### 2.7. Model development and internal validation

Three logistic regression prediction models were constructed: a radiomics-only model using the LASSO-selected multisequence features; a clinical plus rehabilitation model; and a combined model integrating the selected radiomics features with clinical variables and rehabilitation adherence.

The dataset was randomly divided in a stratified 70/30 ratio into training and validation sets to preserve the recurrence proportion. All preprocessing and feature selection were confined to the training data, and the fitted transformations and coefficients were then applied to the held-out validation data. Hyperparameters were tuned by grid search with 10-fold cross-validation within the training set. The single split was selected as a transparent internal-validation design for this dataset; however, its estimates remain sensitive to partition variability. Repeated or nested cross-validation and independent external validation are needed to quantify optimism more robustly.

### 2.8. Model performance evaluation and clinical utility

Model discrimination was assessed using receiver operating characteristic curves and the area under the curve (AUC). Accuracy, sensitivity, and specificity were derived from confusion matrices. Model calibration was evaluated using calibration curves generated via 1000-bootstrap resampling. Clinical utility was examined using decision curve analysis, which quantified net clinical benefit across different threshold probabilities.

Patients were stratified into low- (<20%), intermediate- (20%–40%), and high-risk (>40%) groups based on predicted probabilities from the combined model, and recurrence rates were compared among groups.

### 2.9. Statistical analysis

Statistical analyses were performed using IBM SPSS Statistics, version 26.0 (IBM Corp.) and R, version 4.2.2 (R Foundation for Statistical Computing). Normality of continuous variables was assessed using the Shapiro–Wilk test. Normally distributed variables are reported as mean ± standard deviation and were compared using independent-samples *t* tests after assessment of variance homogeneity; categorical variables are reported as counts and percentages and were compared using chi-square or Fisher’s exact tests, as appropriate. Multicollinearity among candidate clinical predictors was assessed using variance-inflation factors, with values <5 considered acceptable. Stepwise multivariable logistic regression was used only as a secondary, clinically interpretable explanatory analysis; high-dimensional radiomics selection was performed separately by LASSO. Model fit and influential observations were assessed using calibration, residual diagnostics, and Cook’s distance. Odds ratios (ORs) with 95% confidence intervals are reported. All tests were 2-sided, and *P* < .05 was considered statistically significant.

## 3. Results

### 3.1. Baseline characteristics

A total of 260 patients with LDH who completed standardized rehabilitation were included, of whom 78 (30.0%) experienced recurrence and 182 (70.0%) did not. Baseline characteristics are summarized in Table 1. Compared with the non-recurrence group, the recurrence group had significantly higher age (50.16 ± 9.18 vs 47.07 ± 9.35 years, *P* = .014), BMI (25.82 ± 2.96 vs 24.83 ± 2.94 kg/m^2^, *P* = .014), and disc height index (0.45 ± 0.07 vs 0.40 ± 0.07, *P* < .001), along with a markedly lower ODI improvement rate (34.58% ± 11.63% vs 49.43% ± 14.03%, *P* < .001). In addition, diabetes (25.64% vs 10.99%, *P* = .005), Modic changes (30.77% vs 15.38%, *P* = .008), and poor rehabilitation adherence (51.28% vs 20.88%, *P* < .001) were more prevalent in the recurrence group. No significant between-group differences were found in sex or smoking history (both *P* > .05).

**Table 1:** Baseline characteristics of patients with and without recurrence.

Variable	Non-recurrence group (n = 182)	Recurrence group (n = 78)	*t*/χ^2^	*P* value
Age (yr)	47.07 ± 9.35	50.16 ± 9.18	−2.475	.014
BMI (kg/m^2^)	24.83 ± 2.94	25.82 ± 2.96	−2.488	.014
Disc height index	0.40 ± 0.07	0.45 ± 0.07	−5.553	<.001
ODI improvement (%)	49.43 ± 14.03	34.58 ± 11.63	8.850	<.001
Male sex	103 (56.59%)	50 (64.10%)	0.980	.322
Smoking	55 (30.22%)	32 (41.03%)	2.399	.121
Diabetes	20 (10.99%)	20 (25.64%)	7.914	.005
Modic changes	28 (15.38%)	24 (30.77%)	7.144	.008
Poor rehabilitation adherence (<70%)	38 (20.88%)	40 (51.28%)	22.607	<.001

χ^2^ = chi-square statistic, BMI = body mass index, n = number of patients, ODI = Oswestry Disability Index.

### 3.2. Radiomics feature selection and importance ranking

**Figure 1.**
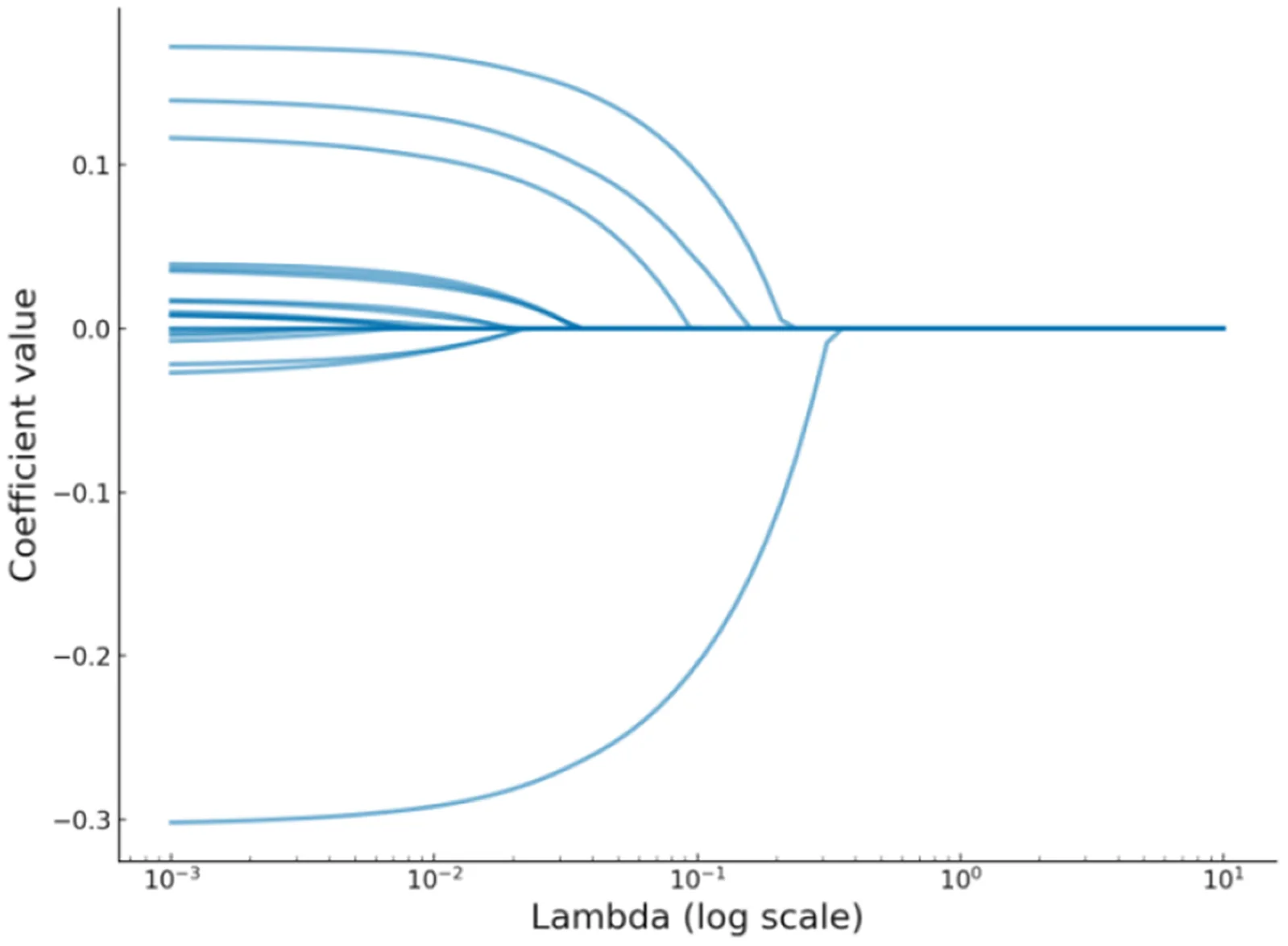
LASSO coefficient path. LASSO = least absolute shrinkage and selection operator.

**Figure 2.**
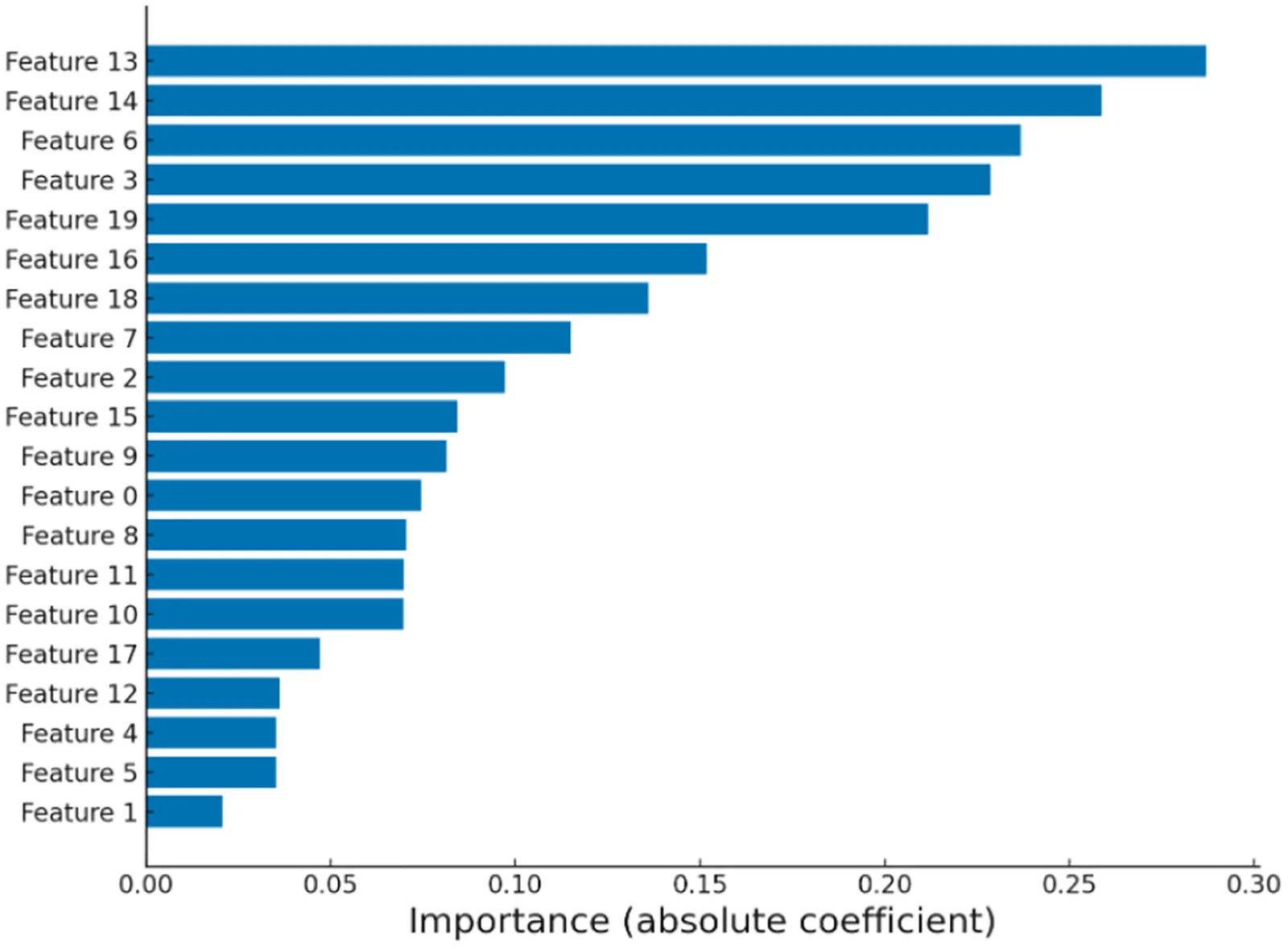
Feature importance ranking.

Radiomics feature dimensionality reduction was performed using LASSO regression. As the penalty coefficient λ increased, most feature coefficients shrank toward 0, leaving only a small number of stable, predictive features (Fig. 1). After integration with clinical variables, feature importance ranking (Fig. 2) revealed that disc height index, ODI improvement rate, and rehabilitation adherence contributed most strongly to recurrence prediction.

### 3.3. Predictive performance of different models

**Figure 3.**
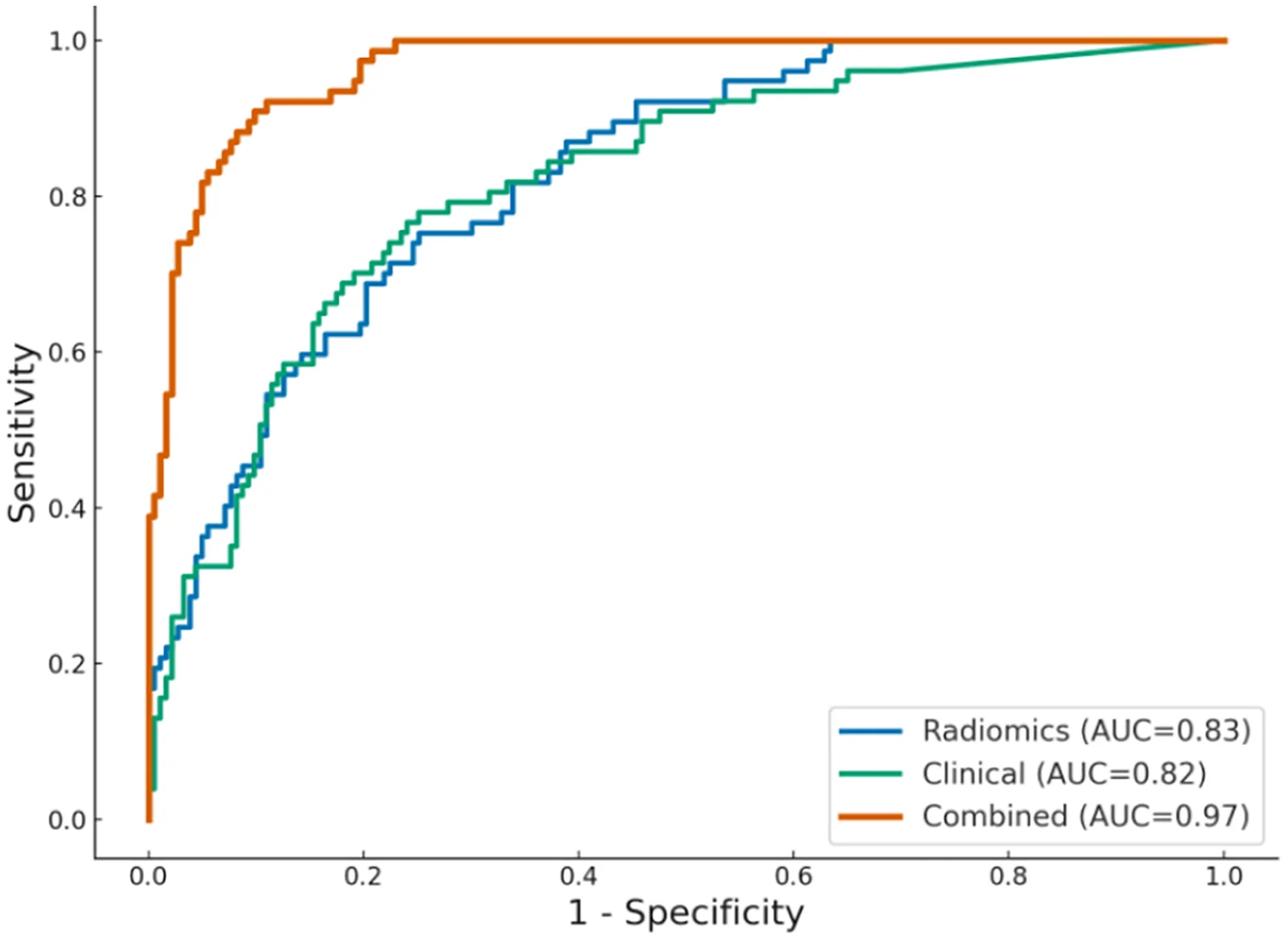
Receiver operating characteristic (ROC) curves for the radiomics, clinical, and combined models. AUC = area under the curve.

Three models were constructed: a radiomics model, a clinical + rehabilitation model, and a combined model incorporating radiomics features, clinical variables, and rehabilitation adherence. Performance comparisons in the validation cohort are shown in Table 2. The combined model demonstrated the highest predictive accuracy, yielding an AUC of 0.85 (95% confidence interval: 0.78–0.91), outperforming the radiomics model (AUC = 0.78) and the clinical + rehabilitation model (AUC = 0.76). Its accuracy, sensitivity, and specificity were 0.82, 0.78, and 0.84, respectively (Fig. 3). These findings indicate that integrating radiomics and clinical information substantially enhances predictive capability.

**Table 2:** Predictive performance of different models in the validation cohort.

Model	AUC	95% CI	Accuracy	Sensitivity	Specificity	Brier score
Radiomics-only model	0.78	0.69–0.86	0.75	0.69	0.78	0.17
Clinical + rehabilitation model	0.76	0.67–0.84	0.74	0.68	0.77	0.18
Combined model (radiomics + clinical + rehabilitation)	0.85	0.78–0.91	0.82	0.78	0.84	0.15

AUC = area under the curve, CI = confidence interval.

### 3.4. Model calibration and clinical decision value

**Figure 4.**
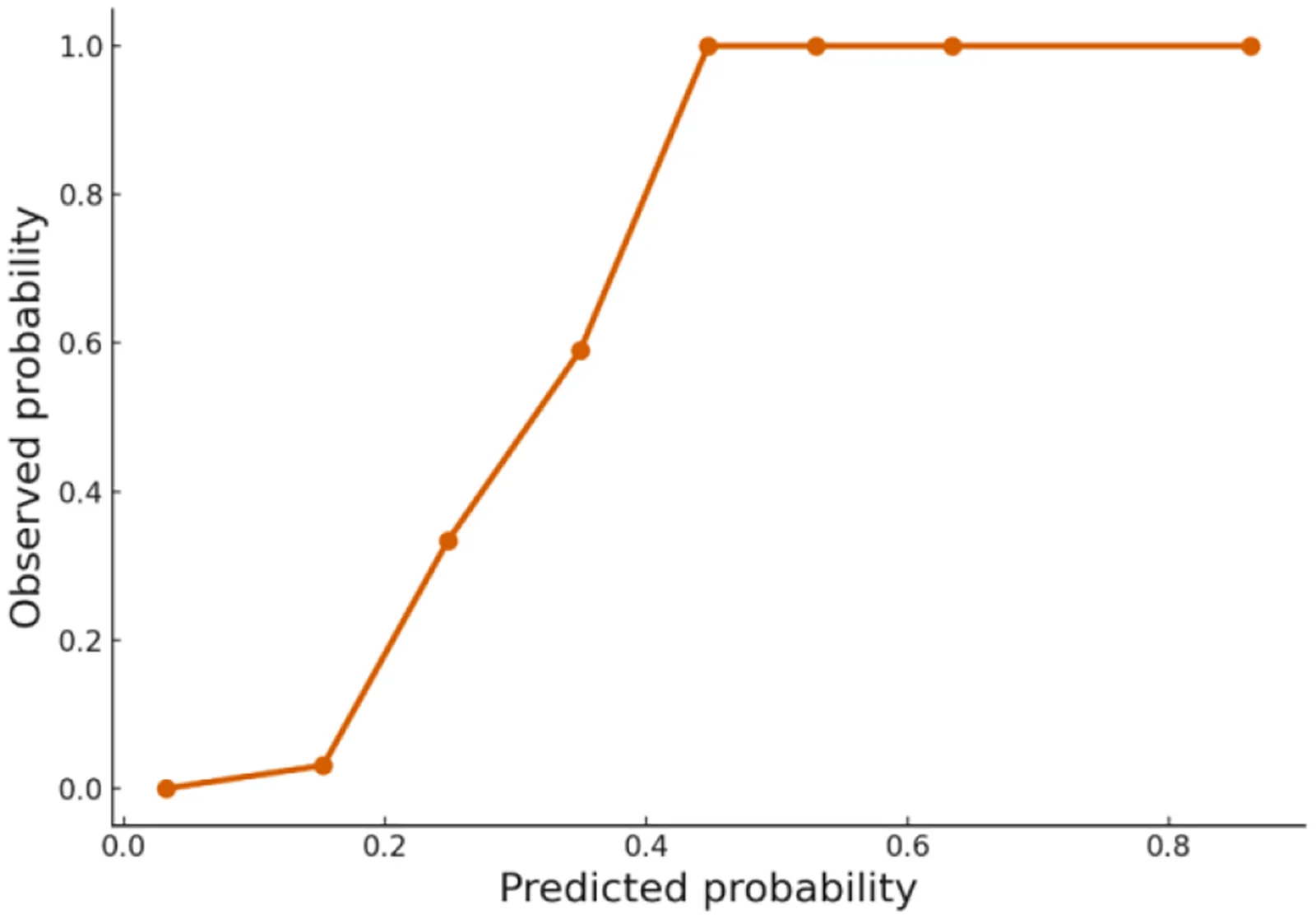
Calibration curve of the combined prediction model.

**Figure 5.**
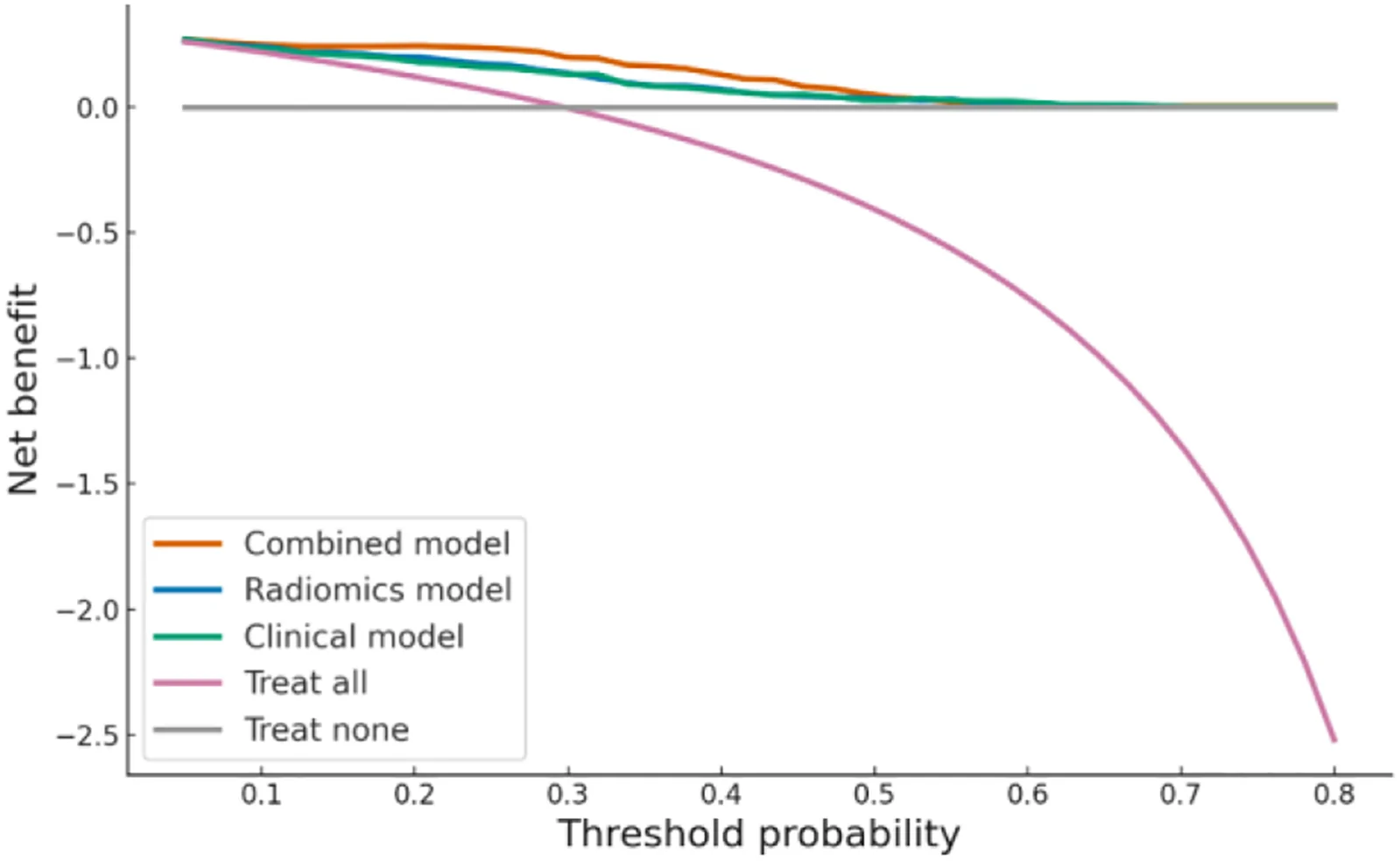
Decision curve analysis of the radiomics, clinical, and combined models.

Calibration analysis of the combined model showed strong agreement between predicted probabilities and observed recurrence rates (Fig. 4). Decision curve analysis demonstrated that across a threshold probability range of approximately 0.10 to 0.70, the combined model provided consistently greater net clinical benefit than the “treat-all” or “treat-none” strategies and outperformed both the radiomics and clinical models (Fig. 5). These results support the combined model’s potential clinical utility for recurrence risk assessment.

### 3.5. Risk stratification performance

Based on predicted probabilities generated by the combined model, patients were stratified into low- (<20%), intermediate- (20%–40%), and high-risk (>40%) groups. Recurrence rates across the 3 groups were 15.0%, 30.0%, and 66.0%, respectively, showing a clear ascending trend (χ^2^ = 43.714, *P* < .001). Pairwise comparisons showed significant differences between all groups (low vs intermediate: *P* = .009; intermediate vs high: *P* < .001; low vs high: *P* < .001; Table 3). This stratification system effectively distinguishes recurrence risk levels and may facilitate individualized clinical management.

**Table 3:** Risk stratification based on the combined model and comparison of recurrence rates.

Risk group	Total (n)	Non-recurrence, n (%)	Recurrence, n (%)	Comparison	χ^2^	*P* value
Low-risk (<20%)	120	102 (85.00)	18 (15.00)	Low vs intermediate	6.818	.009
Intermediate-risk (20%–40%)	90	63 (70.00)	27 (30.00)	Intermediate vs high	14.779	<.001
High-risk (>40%)	50	17 (34.00)	33 (66.00)	Low vs high	43.281	<.001
Overall comparison	–	–	–	All groups	43.714	<.001

χ^2^ = chi-square statistic, n = number of patients, vs = versus.

### 3.6. Multivariable logistic regression analysis

Multivariable logistic regression results are presented in Table 4. Disc height index (OR = 1.80, *P* < .001), ODI improvement rate (OR = 0.65, *P* < .001), poor rehabilitation adherence (OR = 2.50, *P* = .001), diabetes (OR = 2.10, *P* = .026), and Modic changes (OR = 1.90, *P* = .034) were identified as independent predictors of recurrence. Although age and BMI differed between groups in univariable analysis, they did not retain independent predictive significance in the multivariable model (both *P* > .05).

**Table 4:** Multivariable logistic regression analysis of factors associated with recurrence.

Variable	Coding/unit	β	OR	95% CI for OR	*P* value
Age	Per 10-yr increase	0.223	1.25	0.96–1.62	.096
BMI	Per 1 kg/m^2^ increase	0.058	1.06	0.97–1.16	.147
Disc height index	Per 0.10 increase	0.588	1.80	1.32–2.46	<.001
ODI improvement (%)	Per 10% increase	−0.431	0.65	0.52–0.80	<.001
Poor rehabilitation adherence (<70%)	1 = yes	0.916	2.50	1.45–4.32	.001

β = regression coefficient, BMI = body mass index, CI = confidence interval, ODI = Oswestry Disability Index, OR = odds ratio.

## 4. Discussion

This study developed and internally validated a model integrating multisequence MRI radiomics, clinical variables, and rehabilitation adherence to predict recurrence after standardized rehabilitation in LDH. The combined model achieved the highest validation AUC (0.85), compared with radiomics-only (0.78) and clinical plus rehabilitation (0.76) models. It also produced clinically ordered risk groups, while the explanatory analysis identified disc height index, ODI improvement, poor rehabilitation adherence, diabetes, and Modic changes as independent predictors. Together, these results suggest that recurrence reflects complementary structural, metabolic, and recovery-related domains rather than any single marker.

The results of this study align with and extend previous research. Prior studies have shown that increased disc height, endplate degeneration, and segmental instability are closely associated with LDH recurrence.^[19,20]^ Insufficient improvement in ODI reflects suboptimal functional recovery and serves as an early warning marker for recurrence.^[21]^ Poor adherence to rehabilitation programs has also been shown to compromise recovery and increase the likelihood of symptom relapse.^[22]^ Diabetes has been associated with accelerated disc degeneration through the accumulation of advanced glycation end products,^[23]^ which supports our findings. Notably, this study also demonstrates that radiomics features capture subtle variations in disc texture, morphological complexity, and intervertebral microstructural changes, providing additional predictive value. These results echo recent advances in radiomics research in spinal disorders, where quantitative imaging features have shown strong potential for outcome prediction.^[11,14,24,25]^

The strong performance of the combined model in this study is supported by underlying pathophysiological mechanisms. Recurrence in LDH is often related to altered biomechanical properties of the disc, incomplete healing of annular tears, persistent inflammation, or renewed compression of neural tissues.^[14]^ Radiomics features quantify texture, grayscale distribution, and shape characteristics of the annulus fibrosus, nucleus pulposus, and adjacent structures, thereby capturing microstructural alterations that are not discernible on conventional MRI. When combined with clinical variables such as BMI and diabetes, as well as rehabilitation-related factors including adherence and ODI improvement, the model provides a comprehensive representation of both structural damage and functional recovery, yielding superior predictive accuracy and interpretability. The risk-stratification system developed in this study may further inform clinical decision-making, such as intensifying core-stability training, prolonging supervised rehabilitation, or recommending closer follow-up for high-risk patients. The positive association between disc height index and recurrence is hypothesis-generating. One possible explanation is that a relatively preserved disc retains more nucleus pulposus volume and intradiscal pressure, maintaining the biomechanical substrate for renewed protrusion, whereas a severely collapsed disc may contain less mobile material. However, the present retrospective data did not directly measure intradiscal pressure, annular healing, or segmental motion. The association should therefore not be interpreted as proof that greater disc height causes recurrence; prospective biomechanical and longitudinal imaging studies are required.

Radiomics may add value because visually similar discs can differ in signal distribution, texture heterogeneity, and shape complexity. These features may indirectly reflect hydration, matrix disorganization, annular disruption, endplate interaction, or inflammatory change, although individual features are not direct histological biomarkers.^[11,14,24,25]^ The gain in AUC from 0.76 for the clinical plus rehabilitation model to 0.85 for the combined model suggests incremental discrimination from quantitative imaging. Calibration and decision-curve findings further indicate that this improvement was not limited to rank ordering: within the observed threshold range, the combined model offered greater estimated net benefit. Nevertheless, clinical superiority should be understood as internal decision-analytic performance, not proven improvement in patient outcomes.

The risk strata provide a practical bridge from prediction to care. Low-risk patients may continue standard rehabilitation and routine follow-up; intermediate-risk patients may benefit from adherence counseling and earlier reassessment; and high-risk patients may warrant supervised core-stability progression, closer symptom monitoring, metabolic risk optimization, and timely repeat imaging when clinically indicated. These actions are proposed applications rather than tested interventions. A prospective impact study should determine whether model-guided management reduces recurrence, resource use, or disability compared with usual care.

Despite its strengths, this study has several limitations. First, this was a single-center retrospective study, which may have introduced selection bias. In particular, all patients were recruited from a convalescent/rehabilitation center and had completed a standardized rehabilitation program, meaning that their clinical characteristics, treatment adherence, follow-up completeness, and disease spectrum may differ from those of general outpatient or hospitalized LDH populations. This may limit the representativeness and generalizability of the model. Second, spectrum bias should also be considered. The prevalence of recurrence, distribution of imaging severity, and rehabilitation-related behavioral patterns in our cohort may not fully reflect those seen in broader clinical settings. As a result, model performance observed in this internally validated cohort may be more optimistic than that achieved in external populations. Therefore, external validation in independent cohorts from different institutions and clinical scenarios is essential before routine clinical application. Third, although dual-observer verification was used, ROI delineation was based on manual segmentation and remains subject to observer-dependent variation. In addition, even though all MRI scans were acquired on the same scanner model, minor variations in acquisition parameters, coil performance, patient positioning, reconstruction settings, and routine workflow may still influence radiomics feature stability. Although image normalization, resampling, and bias-field correction were performed to improve reproducibility, such preprocessing cannot completely eliminate scanner- or protocol-related variability. Fourth, we did not perform phantom-based harmonization experiments, test-retest reproducibility analyses, or multicenter external validation across different MRI platforms. Therefore, the robustness and transportability of the extracted radiomics features and the final model remain to be established. Future studies should incorporate multicenter prospective cohorts, harmonized imaging protocols, reproducibility assessment, and external validation to strengthen the clinical applicability of this predictive framework. Finally, additional biomechanical or biological markers, such as paraspinal muscle quality, inflammatory biomarkers, or gait-related parameters, were not included in the present study and may further improve model performance in future work.^[26]^

## 5. Conclusion

In this internally validated retrospective cohort, a combined model integrating multisequence MRI radiomics, clinical factors, and rehabilitation adherence discriminated post-rehabilitation LDH recurrence better than either single-domain model. Disc height index, limited ODI improvement, poor adherence, diabetes, and Modic changes identified clinically relevant risk domains, and the derived strata may help clinicians tailor supervision and follow-up intensity. The study’s distinctive contribution is the integration of quantitative tissue characteristics with functional recovery behavior. Before routine use, the model requires prospective multicenter external validation, assessment of reproducibility across MRI platforms, and evaluation of whether model-guided care improves patient outcomes.

## Author contributions

**Conceptualization:** Yanhong Qi, Yifei Feng, Xiaoyue Xing, Zhe Wang, Yuwei Yang, Weiguo Yu.

**Data curation:** Yanhong Qi, Yifei Feng, Xiaoyue Xing, Zhe Wang, Yuwei Yang, Lin Zhang, Weiguo Yu.

**Formal analysis:** Yanhong Qi, Yifei Feng, Xiaoyue Xing, Zhe Wang, Lin Zhang, Weiguo Yu.

**Investigation:** Weiguo Yu.

**Funding acquisition:** Yanhong Qi, Lin Zhang, Weiguo Yu.

**Writing – original draft:** Yanhong Qi, Yuwei Yang, Weiguo Yu.

**Writing – review & editing:** Yanhong Qi, Yuwei Yang, Weiguo Yu.
